# Suspected Pericardial Tuberculosis Revealed as an Amyloid Pericardial Mass

**DOI:** 10.1155/2018/8606430

**Published:** 2018-10-17

**Authors:** Sebastiano Cicco, Antonio G. Solimando, Patrizia Leone, Stefano Battaglia, Roberto Ria, Angelo Vacca, Vito Racanelli

**Affiliations:** Department of Biomedical Sciences and Human Oncology, Section of Internal Medicine “G. Baccelli”, University of Bari Aldo Moro Medical School, Bari, Italy

## Abstract

Primary systemic amyloidosis is not easily diagnosed. The immunoglobulin deposits are usually localized in the kidney, heart, and liver. We describe an unusual case of a patient suffering from a pericardial amyloidoma with internal calcifications and air bubbles that compressed the right ventricle and shifted the heart to the left. Since the patient was in shock, urgent pericardiotomy was performed. This site showed PET uptake. A monoclonal component was present. On these findings, differential diagnoses included multiple myeloma and atypical pericardial tuberculosis, whereas a periumbilical fat tissue biopsy demonstrated amyloidosis. A previous *Salmonella* species infection had most likely stimulated the production of amyloid. The patient received bortezomib/dexamethasone treatment and achieved a good response.

## 1. Introduction

Primary systemic amyloidosis (or AL amyloidosis) is a rare disorder in which misfolded parts of immunoglobulins (usually a monoclonal light-chain fragment) form insoluble fibrillar deposits in organs and tissues [1–6]. Amyloidosis should be suspected in any patient with monoclonal gammopathy and unexplained shortness of breath, fatigue, edema, weight loss, or paresthesias. However, the clinician needs to be well aware of this rare condition because various different symptoms may be present, mimicking more common disorders.

## 2. Case Presentation

A 69-year-old Caucasian man was admitted to our Unit from the Emergency Department. He had been suffering from fever, dyspnea, fatigue, and dizziness for 10 days. He had no relevant medical history until the previous month, when he developed intermittent fever with chills. Ceftriaxone was administered at home without benefit. A few days before hospitalization, the patient's clinical condition worsened. On admission, the patient was confused, jaundiced, and had lower-limb edema.

Physical examination revealed jugular turgor, thready pulse with tachycardia, and hypotension (heart rate, 110 beats/minute; blood pressure, 90/60 mmHg) (Beck's triad). Chest auscultation revealed tachypnea (respiratory rate, 28 breaths/minute) and bilateral basal crepitations. Abdominal palpation disclosed hepatomegaly, splenomegaly, and a dull percussion sound in the lower abdominal quadrants.

Routine blood tests showed elevated white blood cells (2146 × 10^3/*μ*L; neutrophils, 91.1%), normal Hb and PLTs, hyperglycemia, hyperbilirubinemia (bilirubin 5.50 mg/dl; 53% direct), and signs of hepatic dysfunction (aspartate aminotransferase (AST) 93 units/L, alanine aminotransferase (ALT) 119 units/L, gamma glutamyl transferase-GGT 285 units/L, serum albumin 2.9 g/dl, and INR 1.81). NT-pro-BNP was increased (2901 pg/ml), whereas cardiac-specific troponin was in the normal range (Table 1).

**Figure 1:**
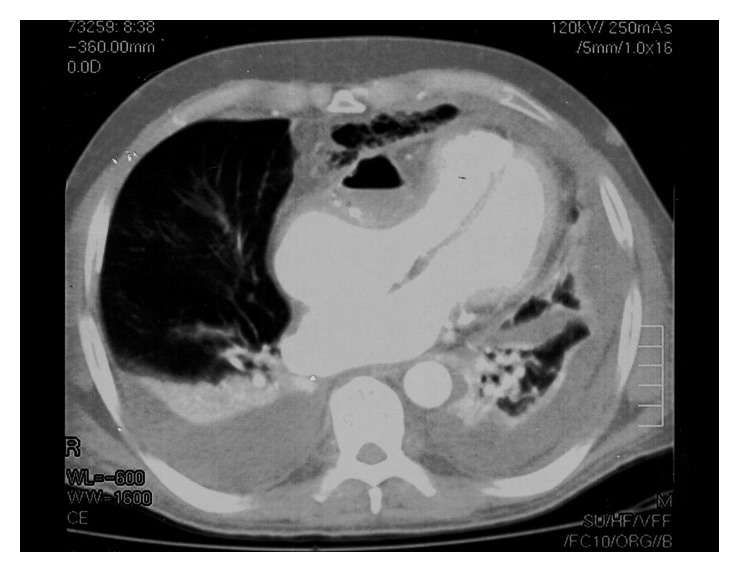
Chest CT scan. The mass is compressing the right ventricle and shifting the heart. Pleural effusion is present, with compression of the left lung.

Since the electrocardiogram (ECG) identified high frequency sites during atrial fibrillation, digoxin and low-molecular-weight heparin (LMWH) were administered. Abdominal ultrasonography (US) revealed signs of hepatic disease (parenchymal inhomogeneity and increased diameters), splenomegaly (longitudinal diameter = 20 cm), and ascites. Heart US showed a difficult contraction and reduced diameter of the right ventricle with right atrium diastolic collapse and no inspiratory changes in the vena cava diameter. Chest CT scan demonstrated a retrosternal mediastinal mass containing calcifications and air bubbles. This mass did not show a cleavage plane over the heart but compressed it and shifted it to the left. Bilateral pleural effusion was also observed (Figure 1). Within a few hours, the blood pressure progressively decreased to 70/40 mmHg; dopamine (7 *µ*g/kg/min) and oxygen (12 L/min) were given, and ECG monitoring was instituted. Urgent pericardiotomy was performed. Constrictive purulent-like pericardium and a firm mass with calcifications were removed. The patient rapidly improved: alertness, arterial pressure, and pulse rate normalized but he still had edema. NT-pro-BNP decreased in a couple of weeks (1430 pg/dl).

Because of the fever, antibiotic treatment was started, and insulin was administered for persistent hyperglycemia. A good response to the therapy was observed and paralleled a reduction of the WBC (11 × 10^3^/*µ*l) and an improvement of liver function, as indicated by a decrease of AST, ALT, and GGT. However, the Hb value (8 g/dl) and PLT count (80 × 10^3^/*µ*l) also decreased, with 3.1% of reticulocytes. Simultaneously, the albumin value was reduced (1.8 g/dl), whereas total bilirubin increased (9.1 mg/dl, 67% indirect), with a positive Coombs test for direct IgG. Therefore, albumin was administered.

One of the possible causes of effusive-constrictive pericarditis is tuberculosis (TBC) [7]. Since negative QuantiFERON ruled out classic TBC, we hypothesized atypical pericardial TBC or a fungal pericarditis. However, all viral (included HIV), microbiological, and cultural tests performed failed to reveal any infections.

**Figure 2:**
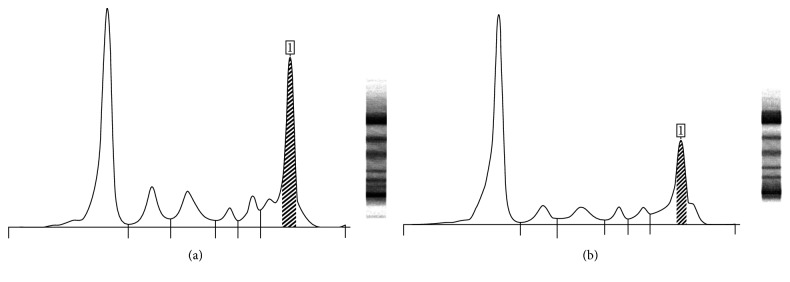
Serum protein electrophoresis before (a) and after (b) therapy. After therapy, there was a reduction of the monoclonal component (1).

Gamma-proteins were increased (30.8% vs 10–20% normal values) on serum protein electrophoresis and contained a monoclonal component (M component) accounting for 23.3% (1.64 g/dl) (Figure 2). Serum immunofixation demonstrated that the M component was IgG-*λ*. Mild renal failure was present with proteins in the urine (1.56 g/24 h) that contained the Bence Jones as free lambda chains. Our analysis showed that the patient had had previous hepatitis A and B infections but not hepatitis C. No bacteria were demonstrated at blood cultures.

**Figure 3:**
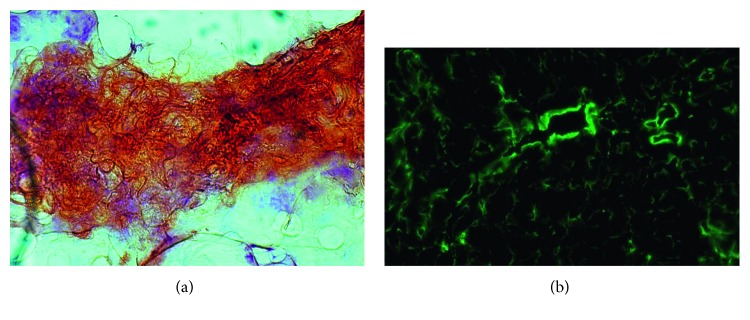
Amyloid deposition in periumbilical fat ((a)-Congo red) with apple-green birefringence (b) at polarized microscope analysis.

The culture test of the pericardial effusion demonstrated the *Salmonella* species (*Salmonella suinis*), so imipenem and ciprofloxacin were given. A sample of periumbilical fat was taken and histologically analyzed: amyloid AL deposits were found as positive Congo red and apple-green birefringent areas under polarized light (Figure 3).

Bone marrow (BM) biopsy showed 4% plasma cells (CD138 and lambda light chain positive) but not amyloid deposits. Skeleton radiological analysis was negative for bone lesions.

PET-CT with 18 fluorodeoxyglucose (FDG) showed a low uptake only in the mediastinum behind the sternum close to the heart. Optical microscope histological examination of the mediastinal mass showed hematoxylin-eosin pink amorphous material which was Congo-red positive and apple-green birefringent and included some granulocytes and few plasma cells (CD138 and lambda light chain positive) near the vessels.

We decided to start treatment according to the chemotherapy scheme VEL/DEX (bortezomib 1.3 mg/m^2^ i.v. on days 1, 4, 8, and 11; dexamethasone 40 mg i.v. on days 1, 2, 4, 5, 8, 9, 11, and 12).

The patient demonstrated good compliance and had a good response. The M component progressively lowered (12.2%, 0.67 g/dl), renal function improved, and urine proteins reduced. Epoetin A treatment was started to prevent a further decrease of Hb values.

At discharge, Hb, PLT, and serum bilirubin were stable; AST and ALT levels were normal; the pleural effusion, dyspnea, tachypnea, and edema had disappeared.

The patient underwent 4 cycles of Vel/Dex yielding a good clinical response. He slowly went back to work, with a good performance status.

## 3. Discussion

Amyloidosis is due to extracellular deposition of insoluble fibrils with a *β*-pleated sheet structure that are formed by the polymerization of misfolded proteins [1–4]. Regardless of the protein type, all amyloid proteins intercalate Congo red dye and show apple-green birefringence under polarized light because they share similar ultrastructural characteristics as seen on electron microscopy [1–6].

Amyloid deposition inside organs progressively interferes with structure and function and results in death. Commonly affected organs include the heart, kidneys, gastrointestinal tract, liver, and peripheral or autonomic nervous system [1–4]. Loss of heart function is the main cause of poor prognosis [5–7]. However, a pericardial extracardiac nodule, as described here, is rare. The amyloidosis deposits may be due to AL amyloidosis or sometimes due to lymphoproliferative disorders such as MALT lymphoma, multiple myeloma (MM), or diseases that involve chronic inflammation [8–16]. Therefore, our patient was subjected to BM biopsy.

Many reports show that the most common site of localized AL amyloidosis is the mucous membranes in contact with the environment [8]. Accordingly, it has been deduced that amyloidogenic plasma cell clones may be triggered by antigenic stimulation. In our patient, the amyloid deposition occurred in a serous membrane: one may tentatively hypothesize that the *Salmonella suinis* infection could have stimulated inflammation and consequent polyclonal immunoglobulin production as well the growth of a plasma cell clone producing the amyloidogenic IgG.

In clinical practice, several diagnostic problems may present. No imaging technique is able to characterize a mass as amyloid deposition. Many authors have described amyloid masses with an uptake activity that can be detected by FDG-PET. This finding could be due to the presence of a tumor and inflammatory cells inside the mass that are detected by FDG because of their increased metabolic activity. Although everyone agrees on the low rate of false positives, these can occur in cases of TBC, aspergillosis, histoplasmosis and inflammatory disorders. Moreover, FDG as a radiotracer may not be sensitive enough to detect amyloid, despite a few reports on the possible utility of FDG in AL amyloidosis, in patients with large masses [17–19].

Amyloid deposits are usually observed within a strictly limited but not encapsulated area. Involvement of vessel walls is common and occurs when vessels lie very close to the main deposit. Abdominal fat biopsy is the advisable choice for the examination of histopathological samples. It is highly informative for AL amyloidosis, featuring 73% sensitivity and 90% specificity, and a low risk of bleeding [20]. AL amyloid can localize in virtually any body site. However, soft tissue deposition is unusual [20–25]. In literature, only few cases of localization of an amyloidoma in the mediastinum, retroperitoneum, or pericardium have been described [25–27]. Those cases were mostly due either to tumor masses or to a previous history of autoimmune disorders, such as rheumatoid arthritis. In all cases, the deposits were in the thymus or in mediastinal lymph nodes [28]. In our patient, none of these previous pathologic conditions were described and the massive amyloid deposits on the pericardium were the only damage triggering the acute symptoms.

AL amyloidosis has a dismal prognosis, with a mean survival of one to two years after diagnosis, and only four months in cases of clinical heart failure if no treatment was performed [2, 3]. Although survival in AL amyloidosis has improved, with a 5-year overall survival rate of 28% of patients, it still remains poor compared to the survival rate of patients with MM (about 40% 5-year survival) [29]. The heart involvement usually determines the prognosis: death often occurs as a result of refractory heart failure or sudden arrhythmic death [1–4]. Cappelli et al. [30] demonstrated that right ventricle damage is of late onset in already known amyloidosis. Conversely, in our case, the right heart was involved at presentation: it was directly compressed by the pericardial amyloid mass. Perhaps, an overall myocardial deposition was also present, which could explain the onset of atrial fibrillation.

Because the pathogenesis of AL amyloidosis overlaps that of other plasma cell dyscrasias, such as MM, the treatment is the same. In our patient, a stem cell transplant was not feasible due to the bad clinical situation and to the high risk related to the procedure [2]. The choice of Vel/Dex therapy, already used in MM, seemed the most suitable. Many studies have demonstrated a good response to this regimen: 71% hematological response and 25% complete response [2–7, 31].

Initial clinical data showed the presence of constrictive pericarditis. One possible cause could be TBC: clinical symptoms in our patient were similar to those usually found in TBC. Pericardial tuberculosis is a rare condition in developed countries, affecting less than 4% of patients with TBC [32]. However, many new cases are arising in our latitude, mostly in HIV patients, immigrants, or in those who work with them [33]. The presence of calcification sustained this idea because it is mostly related to a long-term inflammation and it suggests a dry/adsorptive stage of tuberculous pericarditis [32]. Other infective conditions leading to a constructive purulent pericarditis are even rarer (<1%) [32].

On the other hand, the presence of the M component raised a differential diagnosis problem. The slow decrease in Hb and PLT values and the increase in WBC count associated with the M component suggested that the pericardial mass was a plasmocytoma, but there were too few plasma cells in the mass. The hepatopathy and ascites were not related to viruses, and a correlation to TBC or MM was difficult to conceive. Amyloid liver deposition could be a good explanation. Moreover, deposits in the pericardium are frequently due to the presence of AL amyloidosis in the lymph nodes. In serous membranes, nontumor-related deposits are rare. However, a pericardial amyloid solitary mass has never been previously described.

We believe that the patient had a previous infection sustained by *Salmonella suinis* that triggered the normal immune response and inflammation. This could explain the fever. Perhaps, an amyloidogenic plasma cell (PC-AL) clone was already present at the time of infection, and its growth may have been stimulated by inflammatory cells. However, we do know how long an amyloid takes to have a production and deposition. The wrong immunoglobulin gene arrangement shifts the antibody production to a massive AL production that misfolds, producing amyloid. Moreover, bacteria inside the mass produce air due to their metabolism. These bubbles, that grow even bigger, participate in the heart compression and sudden heart tamponade. The presence of PCs inside the mass could be a good explanation of the FDG intake near the heart.

In conclusion, in AL amyloidosis, the diagnosis is commonly difficult. Many other diseases show a similar presentation. In our patient, TBC and MM could explain just a part of the symptoms. The fat-tissue biopsy result, related to the clinical data, ultimately suggested another possible yet still unknown presentation of amyloidosis. This case remains a paradigm of the clinical challenge that physicians have to face. In fact, many confounding factors are present together; so many different specialties need to be involved. In a nebulous clinical setting, a rare disease could be an explanation. After considering life-threatening conditions, related to the evidence-based approach, we quickly need to explore the possible differential diagnoses starting from the most frequent conditions. The unexpected finding of a monoclonal component drives a hematological approach in an apparently infective setting. This case should teach us never to exclude even the rarest conditions. In fact, due to the clinical presentation, amyloidosis was a really unexpected result, while the purulent pericarditis was a concomitant disease.

## Figures and Tables

**Table 1:** Routine laboratory investigations.

Value	Admission	After surgery	
Hb	13.4	8.0	g/dl
MCV	60.0	62.3	fl
RBC	7.31	4.36	×10^6^/*μ*l
WBC	21.46	11.12	×10^3^/*μ*l
Neutrophils	91.1	91.6	%
Leucocytes	3.6	3.7	%
Monocytes	4.6	4.1	%
Eosinophils	0.3	0.3	%
Basophils	0.5	0.3	%
Platelets	132	80	×10^3^/*μ*l
Creatinine	1.44	1.85	mg/dl
BUN	46	68	mg/dl
Na^+^	148	135	mmol/l
K^+^	4.0	3.4	mmol/l
Cl^−^	112		mmol/l
Ca^++^	8.4		mg/dl
Glucose	152		mg/dl
Tot. bil.	5.5	9.10	mg/dl
Direct	3.5	5.20	mg/dl
AST	93 (×3.1)	Norm	Units/l
ALT	119 (×1.6)	Norm	Units/l
GGT	285 (×3.3)	Norm	Units/l
Tot. prot.	6.7		g/dl
Albumin	2.9	1.8	g/dl
Amylase	23		U/l
Lipase	190		U/l
CRP	132	195	mg/l
PT INR	1.81	1.79	
aPTT	1.05		
Fibrinogen	368	405	Mg/dl
cTnI	<0.01		ng/ml
NT-pro-BNP	2901	1430	pg/ml
Quantiferon	Negative	Negative	
Blood cultures	Negative	Negative	
